# Enhancing fake news detection with transformer-based deep learning: A multidisciplinary approach

**DOI:** 10.1371/journal.pone.0330954

**Published:** 2025-09-09

**Authors:** Nabeel Raza, Said Jadid Abdulkadir, Yawar Abbas Abid, Sami S. Albouq, Ayed Alwadain, Asad Ur Rehman, Ebrahim Hamid Sumiea, Muhammad Farhan

**Affiliations:** 1 Science Island Branch Graduate School University of Science and Technology Hefei, Anhui, China; 2 Department of Computer and Information Sciences, Universiti Teknologi PETRONAS, Seri Iskandar, Perak, Malaysia; 3 Department of Computer and Information Systems, Islamic University of Madinah, Medina, Saudi Arabia; 4 Center for Research in Data Science (CeRDaS), Universiti Teknologi PETRONAS, Seri Iskandar, Perak, Malaysia; 5 Department of Computer Science, COMSATS University Islamabad, Sahiwal, Pakistan; 6 School of Computer Science and Information Security, Guilin University of Electronic Technology, Guilin, China; 7 Department of Computer Science, Community College, King Saud University, Riyadh, Saudi Arabia; 8 School of Cyber Science Engineering, Wuhan University, Wuhan, Hubei, China; Majmaah University, SAUDI ARABIA

## Abstract

The widespread dissemination of fake news presents a critical challenge to the integrity of digital information and erodes public trust. This urgent problem necessitates the development of sophisticated and reliable automated detection mechanisms. This study addresses this gap by proposing a robust fake news detection framework centred on a transformer-based architecture. Our primary contribution is the application of the Bidirectional Encoder Representations from Transformers (BERT) model, uniquely enhanced with a progressive training methodology that allows the model to incrementally learn and refine its understanding of the linguistic nuances that differentiate factual reporting from fabricated content. The framework was rigorously trained and evaluated on the large-scale WELFake dataset, comprising 72,134 articles. Our findings demonstrate the model’s exceptional performance, achieving an accuracy of 95.3%, an F1-score of 0.953, precision of 0.952, and recall of 0.954. Comparative analysis confirms that our approach significantly outperforms traditional machine learning classifiers and other standard transformer-based implementations, highlighting its superior ability to capture complex contextual dependencies. These results underscore the efficacy of our enhanced BERT framework as a powerful and scalable solution in the ongoing fight against digital misinformation.

## 1 Introduction

In the contemporary digital ecosystem, the proliferation of “fake news”, verifiably false information presented as authentic news, has emerged as a critical threat to societal well-being, public discourse, and democratic processes [1]. The speed and scale at which misinformation can spread through social media and other online platforms can sway public opinion, incite unrest, and erode trust in established institutions [4]. Consequently, the development of automated, accurate, and robust methods for detecting fake news is not merely a technical challenge but a societal imperative [3].

Early attempts to combat fake news primarily relied on traditional machine learning models using surface-level linguistic features. While useful, these methods often fail to capture the subtle semantic and contextual nuances that distinguish sophisticatedly crafted fake news from genuine reporting [6,7]. More recent approaches have leveraged deep learning, but many still depend heavily on metadata, such as user engagement patterns or source credibility scores [8,9]. Such dependencies limit a model’s applicability, as this metadata is often unavailable, easily manipulated, or absent in the early stages of a news item’s lifecycle.

The advent of transformer-based models, particularly the Bidirectional Encoder Representations from Transformers (BERT) model, marked a paradigm shift in natural language processing (NLP) [5]. By processing the entire context of a word simultaneously, BERT can capture deep, bidirectional relationships within text, making it exceptionally powerful for text classification tasks. However, simply fine-tuning a standard BERT model for fake news detection does not guarantee optimal performance. The heterogeneous and complex nature of fake news text requires a more specialized approach to unlock the model’s full potential. We addressed the research question: Can a progressive, episode-based training methodology significantly improve the performance and robustness of a BERT-based model for fake news detection when compared to a standard, single-run fine-tuning approach.

To address this gap, this study introduces a novel fake news detection framework whose primary innovation lies in a progressive training methodology applied to the BERT model. Instead of a single, monolithic training session, our approach iteratively trains the model in stages, allowing it to learn foundational patterns first before refining its understanding on more complex examples. This progressive technique enhances the model’s robustness and improves its ability to generalize from the training data to unseen articles. By focusing exclusively on the textual content of news articles, our framework provides a versatile and powerful solution that is not reliant on external or often-missing metadata. The primary contributions of this work are threefold:

1. We propose a novel fake news detection framework featuring a BERT model enhanced with a progressive training strategy to improve classification accuracy and model robustness.
2. We conduct a comprehensive evaluation of our framework on the large-scale and challenging WELFake dataset, demonstrating its state-of-the-art performance.
3. We provide a detailed comparative analysis against standard BERT implementations and other baseline models, empirically validating the superiority of our proposed methodology.

This paper is organized as follows: Sect 2 reviews related works in fake news detection. Sect 3 details our proposed methodology. Sect 4 presents and discusses the experimental results, and Sect 5 concludes the paper with directions for future work.

## 2 Related works

Chen *et al*. [11] outperforms current techniques and suggests a transformer-based language model strategy for identifying COVID-19-related fake news. The authors discuss their strategy, which involves adversarial training, expanding token vocabulary, adapting heated-up softmax loss, merging high-level and fine-grained specialized representations, and emphasizing the importance of domain-specific knowledge and annotated data. Ding *et al*. [12] addresses the difficulties of early detection and limited labelled data in fake news identification by proposing a transformer-based technique. The suggested framework delivers improved accuracy in identifying fake news within minutes of transmission by merging data from news articles and social media. The authors clearly outline their methodology and experimental setup in this well-written study. However, the research does not discuss the potential biases and restrictions of employing social contexts for fake news detection. The paper contributes significantly to the field of fake news detection overall.

Potluri *et al*. [13] present a two-stage pipeline employing machine learning models for COVID-19 fake news identification. Using a fact-checking algorithm, the first step obtains information, and the second stage uses a manually curated dataset to compute the claim’s truth level. The authors emphasize the value of quick identification to stop the spread of hazardous claims during the pandemic. According to the article, a pipeline built on BERT and ALBERT produces the greatest outcomes. The work does not address the potential biases and restrictions of utilizing a manually curated dataset.

Al-Yahya *et al*. [14] compare linguistic models based on transformers and neural networks for Arabic Fake News Detection (FND). Despite the several methods for Arabic FND that have been suggested, most of them ignore current developments in natural language processing. The authors consider the neural networks. Language models that are based on transformers and compare their functionality. According to the outcomes, transformer-based models are more efficacious than the solutions based on the neural networks, enhanced the accuracy of 16% percent and increased the F1 score of 0.83 to 0.95. The publication also marks the gaps in the research on Arabic FND and suggests the opportunities to further research.

According to the study by Gundapu *et al*. [15], false news about coronavirus disease can be tracked with the help of the following method. merging three models of transformers. To impede panic and uncertainty, in order to prevent it, we have to stop panic and uncertainty, there wartime in order to find power in numbers, which has spread throughout the pandemic, the article notes how the early fake news detection. According to the authors, their approach is good yielding accuracy. came fifth out of 160 groups on the joint Constraint AI 2021 task. Besides this, the physical culture itself is something beyond its natural state. which emphasize the worth of using transformer models to detect fake news. research underlines the necessity to solve the issue of fake news about the COVID-19. Social media sites. According to Verma *et al*. [16], one can apply the M Cred framework. determine false news on social media. As regards global and local text semantics, respectively, the The framework we are using has BERT and CNN. The authors demonstrate that M Cred works. Improved 1.10% over the best models on a Kaggle dataset and reiterate the worth of employing both local and global semantics of the text in the detection of fake news. The paper shows the efficiency of the proposed structure along with BERT and CNN. Glazkov *et al*. The article by [17]. introduces a procedure of detecting fake news about COVID-19 on social media based on the use of a family of COVID-Twitter-BERT (CT-BERT) transformer based models. The Of the 166 participated teams in the Constraint@AAAI2021 Shared Task, the method ranked first. good test set F1-score of 98.69. So that false information should not spread further due to panic and lack of knowledge in the event of the pandemic, the case study outlines the importance of identifying fake news. news and shows the performance of CT-BERT and ensemble learning at it. Raza *et al*. [18]. operates on a Transformer structure and the data of news articles. Social situations that are used to offer a platform of defying false information associated with the Twitter and 2020 US presidential election. The proposed solution finds fake news at a much earlier stage and is more accurate than baseline models. The research points out the effectiveness of Transformer-based fake news detection strategy. The relevance of preventing of false information spread on political forums. Li *et al*. [19] describe their work on the COVID-19 Fake News Detection in English shared task in AAAI 2021 where they received rank 3 with weighted F1 score of 0.9859 on test. Among others, training techniques such as warm-ups, learning rates scheduling and k-fold cross-provision, and language models they recommended, just to mention a few, include, BERT, Roberta and Ernie pre-trained models into an ensemble method. Noticeably, the percentage of the incorrectly sampled types is also carefully analyzed in the study.

Fawaid *et al*. [34] examine fake news in Indonesia with an emphasis on detection and prevention in Bahasa Indonesia. Different models were examined using a dataset of 1116 news articles mixed with other datasets. The Transformer Network and Bert method’s 90% accuracy demonstrates its potency. The study assessed the attack resilience of various false news detection techniques, exposing weaknesses, particularly in the visual domain. To increase robustness, defensive methods were suggested, highlighting the value of real-world testing. Zhou *et al*. [21] provide a novel technique for identifying bogus news based on the linguistic style known as the hierarchical recursive neural network (HERO). The suggested method creates a hierarchical linguistic tree of news stories to capture the writer’s word choice and recursive structure, improving classification accuracy on real-world datasets. As part of its investigation into how to recognize false news and create strategies to resist it, the article also compares the linguistic styles of the two types of information.

Bounaama *et al*. [22] including sentiment analysis and fake news detection during COVID-19. They used NLP techniques and BERT to obtain good accuracy using the “task1.c” dataset, which contains 4128 sentiment analysis tweets and 8661 false news tweets. Results: 90% accuracy in identifying false news; 93% accuracy in analyzing sentiment. shows how effective pre-trained language models are for analyzing social media in real-time. Suryavardan *et al*. [23] introduce a brand-new multi-modal dataset for automatically vetting satirical and false news for accuracy. The dataset has 50,000 instances in three major categories, are textual and graphic, and covers both. The paper also provides a baseline model using BERT and applying vision transformer that is well performing on the test set achieving 65 % F1 on the test set. The new proposed data and model is expected to assist in combating fake news and satirical news in addition to improving automation fact-checking approaches.

Jiang *et al*. [24] propose the novel approach to detect false messages basing on multimodal information. The disadvantage of the traditional methods is that noise is added to the features and a substantial amount of training cases are required. To solve these issues, the study proposes a system, Similarity-Aware Multimodal Prompt Learning (SAMPLE), which entails prompt learning and the application of similarity-aware fusing technique to minimize noise injection. The proposed approach outperforms previous studies on two benchmark datasets and can be a practical application usable in a real-world scenario. Heidari *et al*. [25] explore the impact of social bots in dissemination of misinformation during the pandemic. According to the authors, brand-new technique based on BERT and transfer-learning will help to recognize bot accounts and improve fake news detection. Although the conclusions are reached on the limited data, the study provides a new insight into bot identification and the detection of fake news on the Internet resources.

Balouchzahi *et al*. [26] discuss the issue of spotting fake news in several fields in the COVID-19 period. For the shared task of fake news detection, which has two subtasks, fake news detection and topical domain classification, the authors explain models created by the team MUCIC. The suggested models combine Roberta, Distilbert, and BERT, three transformer-based language models, for fine-tuning, and use majority voting. For Subtasks 3A and 3B, respectively, the models received F1-scores of 0.5309 and 0.8550. Babu *et al*. [27] examine how well pre-trained transformer-based language models like BERT, Roberta, ALBERT, and Distil BERT perform on three false news datasets. Roberta consistently outperformed the other models in the experiments, and Distil BERT trained more quickly than the others. The report highlights the significance of expanding fake news detection research and offers insightful information to the research community.

Pritzkau *et al*. [28] “Multi-class fake news detection of news articles and domain identification with Roberta - a baseline model” contains the strategy adopted by the team of NLytics in the fields of multi-class classification issue of None to detect fake texts. The Roberta is an architecture that was picked to do sequence classification and it was better on the data provided with annotations that were used in supervised training sessions. The report does not provide concrete numbers or comparisons; it is a kind of a benchmark model of further research in the identification of false news. The work by Nasser *et al*. [29] proposes a two-step approach of defining brief fake news on social media based on BERT. The model relies on available information to add features and compute attention weights, as well as identify false news, as a fine-grained multi-classification task. The model is evaluated with a benchmark data coming out of the real world and has been seen to outperform on the baseline as well as other methods enhancing the present body of research on the identification of fake news.

Silva *et al*. [30] respond to the unavailability of labeled data that are not in the English language and the rising spread of malicious information on social media. It provides practical means of automatic false news detection in Portuguese by testing with several properties and classification methods. Through this work, the study in the field will progress, and light will be shed on problems revolving around detecting false news in non-English languages. Fake news detection methods are rated in terms of the four perspectives of inappropriate understanding, writing style, pattern of transmission, and credibility of sources in Zhou *et al*. [2]. It underlines the importance of interdisciplinarity and points to some major theories of different disciplines. Through survey, the Faculty of Journalism, Faculty of Political Science, Faculty of Social Sciences and Faculty of Computer Science specialists are urged to collaborate in developing effective and easily intelligible methods of detecting false news. It provides the suggestions to the idea of following field research work and promotes existing researches.

A new approach to detecting fake news on social media, developed by Hu *et al*. [31], is quite ingenious. The authors present a framework known as the TriFN framework that consists of modeling the interaction between news items, publishers, and users and can be used to enhance the identification of false news. The given approach showed superior results in comparison with other conventional approaches when it came to real-world datasets. The study helps fill the gap in the archive of studies dealing with this topic by indicating the importance of considering the social context when identifying false news to be more precise. EEG-based attention tracking is in the spotlight in adaptive learning systems in past years [32]. As an example, Rehman *et al*. (2024) offered an original mechanism based on an EEG signal that identifies the attention state of a learner in an online learning scenario that deals with a Double Deep Q-Network (DDQN). They succeeded in finding the changing attention levels and their pattern with great high rates as compared with preceded models. Knowledge advances of this nature are significant components of the increasing significance of real time neural based feedback mechanisms in the optimization of the digital learning platforms.

Liu *et al*. [33] propose to apply the use of FNED, a deep learning network, in the detection of fake news in the social media prior to propagation. The limited instances of early-stage data can however be effectively used by FNED to perform above the baselines. This model has very high precision in identifying fake news using minimal labeled data and it has practically offered solutions to the fake news problem on social media as demonstrated by Table 1.

**Table 1:** Comparison of research papers on various topics, highlighting their approach, key contributions, and other evaluation matrices.

Reference	Methodology and Approach	Evaluation and Performance
Chen *et al*. 2021 [11]	Transformer-based language model fine-tuning	Best-weighted average F1 score of 9
Raza *et al*. 2022 [12]	Transformer with encoder/decoder, news, and social context	Higher accuracy than baselines
Vijjali *et al*. 2020 [13]	Two-stage automated pipeline	Best results from pipeline based on BERT and ALBERT
Al-Yahya *et al*. 2021 [14]	Transformer-based language models and neural networks	For the best transformer-based model, F1 scored 0.95
Gundapu *et al*. 2021 [15]	Three transformer models (BERT, ALBERT, and XLNET) combined	F1-score of 0.9855 on test set, placing fifth out of 160 teams
Verma *et al*. 2022 [44]	Message Credibility	Experimental results on a Kaggle dataset
Glazkova *et al*. 2021 [17]	Ensemble of COVID-Twitter-BERT (CT-BERT) models based on transformers	Obtained a weighted F1 score on the test set of 98.69
Raza *et al*. 2021 [18]	Novel framework using Transformer architecture	Experimental results on real-world data
Li *et al*. 2021 [19]	Different pre-trained language models together in an ensemble	Obtained a weighted F1 score on the test set of 0.9859
Fawaid *et al*. 2021 [34]	BiLSTM, CNN, and Hybrid BERT with Transformer Network on CNN-BiLSTM	90% accuracy is achieved using the BERT technique and the Transformer Network
Chen *et al*. 2023 [20]	Simulation of adversarial and backdoor attacks	Proposed robust evaluation of fake news detectors
Zhou *et al*. 2023 [21]	HERO (hierarchical recursive neural network)	HERO beats current methods in news document classification
Bounaama *et al*. 2023 [22]	Natural language processing tools, BERT	0.93 (sentiment analysis), 0.90 (fake news detection)
Suryavardan *et al*. 2023 [23]	BERT and Vision Transformer	65%
Jiang *et al*. 2023 [24]	SAMPLE framework for multimodal prompt learning	Outperforms prior works in F1 and accuracy, regardless of data scarcity
Heidari *et al*. 2021 [25]	Transformers’ Bidirectional Encoder Representations (BERT)	New algorithm for detecting fake news and bots in the COVID-19 dataset
Fazlourrahman *et al*. 2021 [26]	Transformers’ Bidirectional Encoder Representations (BERT)	Dataset for COVID-19
Babu *et al*. 2023 [27]	Roberta, DistilBERT, and BERT from Hugging-Face	F1-scores: 0.5309 (Subtask 3A) and 0.8550 (Subtask 3B)
Pritzkau *et al*. 2021 [28]	BERT, RoBERTa, ALBERT, and DistilBERT	RoBERTa: 69% accuracy on LIAR dataset, DistilBERT trained in 3.5 mins
Liu *et al*. 2019 [?]	Two-stage model built on BERT, fine-grained sentiment analysis	Detecting bogus news with effectiveness and fine-grainedness
Silva *et al*. 2020 [30]	ML methods for fake news detection analysis	Shedding light on the technologies’ potential and the difficulties false news detection poses
Zhou *et al*. 2020 [2]	False knowledge, writing style, transmission patterns, and source reliability	Evaluating fake news detection methods from 4 perspectives, identifying research tasks and theories across disciplines
Nguyen *et al*. 2019 [46]	Framework for integrating three relationships TriFN	Superior to other baseline techniques for fake news detection
Liu *et al*. 2020 [33]	Crowd response-based DNN with attention and pooling mechanisms	Achieving greater than 90% accuracy for early detection of fake news

## 3 BERT-based framework for effective fake news detection

**Fig 1:**
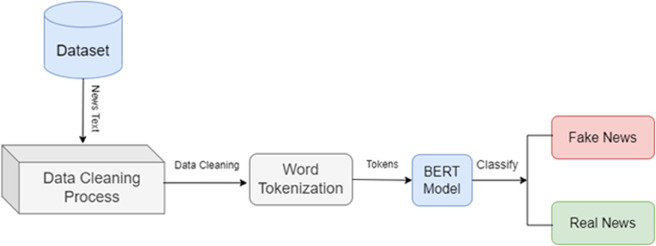
Proposed framework for content-based fake news classification.

The framework of the methodology of fake news detection implies starting with exploratory data analysis (EDA), i.e., the analysis of the dataset upon which the task is performed to receive information about the characteristics of the dataset. This involves analysis of data distribution, loss of classes and data quality. They use descriptive statistics and visualizations as well as other applicable methods to conceptualize the characteristics of the dataset with the aim of determining which patterns and/or anomalies can be affecting its performance in terms of the model. The methodology framework is highly critical in terms of data cleaning. To guarantee quality and consistency of the dataset, the pre-processing of the dataset has been carried out. This can include managing missing values and this can be as a result of bad data or incomplete data. The preprocessing the text data is also accomplished through text cleaning techniques. This may include removal of special characters, constant lower-casing of characters and stemming/lemmatization techniques to decompose words to a position of their roots. The elimination of the stop words can also be applied to eliminate words such as the, and, and in which cannot be helpful in detecting the fake news. In order to prepare the dataset to be used in further stages of processing and training the model, a number of data-cleaning operations are implemented. The suggested content-based framework of fake news typology is presented in Fig 1.

The working of the BERT Transformer model involves several equations and operations. The self-attention mechanism computes attention scores between each token in the input sequence, allowing tokens to attend to other tokens. Given an input sequence of tokens, the self-attention mechanism computes a set of attention scores, which are used to weight the importance of different tokens as shown in Eq (1).

$$\text{Attention}(Q,K,V)=\text{softmax}\left(\frac{QK^{T}}{\sqrt{d_{k}}}\right)V$$ (1)

Where *Q*, *K*, and *V* are matrices obtained by multiplying the input token embedding by learnable weight matrices. The softmax function normalizes the attention scores, and *d*_*k*_ represents the dimensionality of the token embedding. BERT employs multiple parallel self-attention layers, called heads, to capture different types of information. Each head learns a separate set of *Q*, *K*, and *V* matrices. The output of the multi-head attention is obtained by concatenating the outputs of all the heads and linearly transforming them as shown in Eqs (2) and (3).

$$\text{MultiHead}(Q,K,V)=\text{Concat}({\text{head}}_{1},\ldots ,{\text{head}}_{h})W^{O}$$ (2)

$${\text{head}}_{i}=\text{Attention}(QW_{i}^{Q},KW_{i}^{K},VW_{i}^{V})$$ (3)

After self-attention, a position-wise feed-forward neural network is applied to each token representation independently. The feed-forward network consists of two linear transformations with a ReLU activation in between, as shown in Eq (4).

$$\text{FFN}(x)=\max(0,xW_{1}+b_{1})W_{2}+b_{2}$$ (4)

Where *x* represents the input token representation, *W*_1_ and *W*_2_ are weight matrices, and *b*_1_ and *b*_2_ are bias vectors. Layer normalization is applied after each sub-layer, which normalizes the activations across the token dimension. It helps in stabilizing the learning process and improving generalization as shown in Eq (5).

$$\text{LayerNorm}(x)=\frac{x-\mu }{\sqrt{\sigma^{2}+\epsilon }}\gamma +\beta$$ (5)

Where *x* is the input, *μ* and $\sigma^{2}$ are the mean and variance along the token dimension, *ε* is a small constant for numerical stability, and *γ* and *β* are learnable scale and shift parameters. To facilitate the flow of information and alleviate the vanishing gradient problem, residual connections are employed. The output of each sub-layer is added to its input as shown in Eq (6).

Output=x+Sublayer(x) (6)

The following phase in the approach framework is model initialization. For natural language processing tasks, such as fake news identification, the BERT (Bidirectional Encoder Representations from Transformers) approach is frequently employed. The transformer’s library BertTokenizer and BertForSequenceClassification classes, which provide the essential resources for tokenizing the input text data and carrying out sequence classification, are used to initialize the BERT model. The foundation model for false news detection is frequently the 124-layer, 1024-hidden, 16-head, 340M parameter, Bert-base-uncased pre-trained model. For binary classification, the number of output labels is set to 2, signifying true and false news. In order to maximize computational efficiency, the model is also set up not to output attentions and hidden states. The to() technique is used to transfer the model to the desired computing device, such as the CPU or GPU, after it has been initialized.

**Fig 2:**
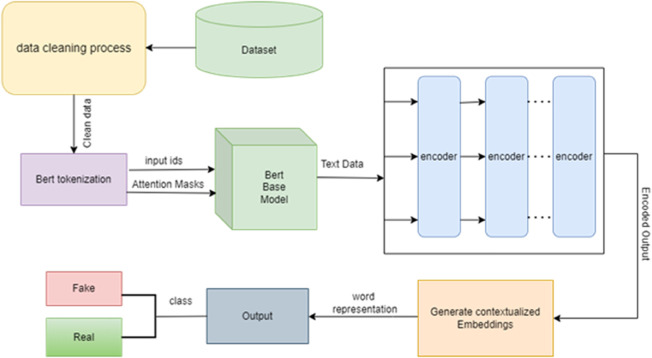
Architecture of proposed methodology.

The data is then batched, divided into smaller subsets, and shuffled to ensure randomness during training, which helps improve the model’s performance and prevents bias towards any particular subset of data. Then, we selected an optimizer and learning rate scheduler and configured them in the methodology framework. The optimizer is responsible for updating the model weights during training. Commonly used optimizers for fake news detection include the Adam or SGD (Stochastic Gradient Descent) optimizer, which adjusts the model weights based on the gradient of the loss function. A learning rate scheduler may also be used to dynamically adjust the learning rate during training for improved convergence. Learning rate is a hyperparameter that controls the step size at which the optimizer updates the model weights. The CosineAnnealingLR or ReduceLROnPlateau schedulers are two often-used learning rate schedulers that modify the learning rate in response to the model’s performance during training. The validation set is used to assess the next step model after it has been trained using the training set. The loss between the predicted outputs and the ground truth labels is calculated after the model has been given input sequences from the training DataLoader. The model weights are then updated using the optimizer depending on the loss. Metrics, including accuracy, loss, F1 score, recall, and precision, are used to track the model’s performance during training and validation. The best-performing model is chosen after comparison and analysis of model performance. The final model is evaluated on the test set following training to determine how well it generalizes. The test set is not used for unbiased evaluation results during model training or validation. measures that are calculated to assess the effectiveness of the model in spotting fake news, including accuracy, F1 score, recall, precision, and other pertinent assessment measures. The architecture of the proposed methodology is shown in Fig 2.

As depicted in Fig 2 The architecture begins with the WELFake Dataset as the primary data source. This data undergoes a rigorous Preprocessing stage, which includes exploratory data analysis, cleaning (such as removing special characters and stop words), and tokenization to prepare the text for the model. The processed data is then fed into the BERT Model, which is fine-tuned using our progressive, episode-based training strategy. The model’s performance is continuously monitored during a Validation phase. Finally, the trained model produces the output, classifying articles as either ‘Real News’ or ‘Fake News’.

**Fig 3:**
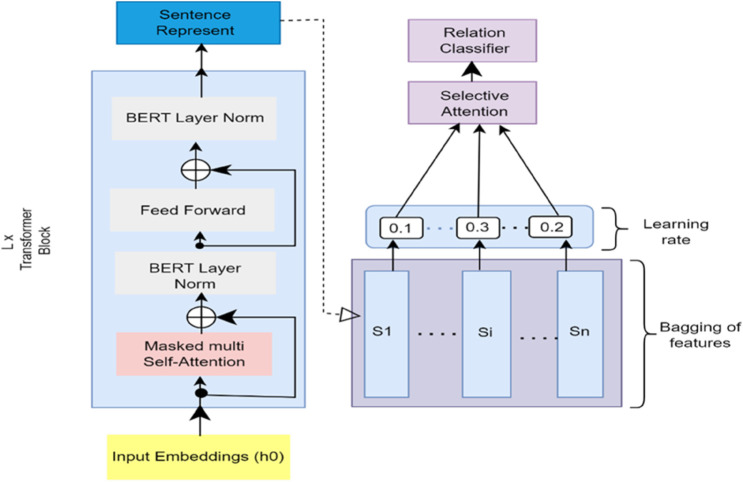
Transformer’s architecture for WelFake fake-news detections.

The Transformer architecture is a powerful deep-learning model introduced in 2017 for various natural language processing tasks. It replaces recurrent neural networks with self-attention mechanisms, enabling the model to effectively capture dependencies between input and output tokens. The encoder and decoder components utilize self-attention and feed-forward networks, with the decoder also incorporating attention over the encoder’s output. Attention mechanisms allow the model to focus on relevant parts of the input sequence. Positional encoding provides positional information without using recurrence or convolution. The Transformer has achieved impressive results in NLP due to its ability to capture long-range dependencies and parallelize computations, making it a foundational architecture in the field. The Transformer architecture is shown in Fig 3.

Fig 3 the typical Transformer architecture is depicted, which becomes the base of our BERT model. In our particular case of fake news recognition, the dataset of the WELFake is fed to the Encoder stack on the left side. In every encoder block, a Multi-Head Attention mechanism enables the model to consider the significance of various words in an article, and it will recognize the contextual relationship that is paramount to detection of deceptive language. The attention layer then outputs data which is salved using a Feed-Forward Network. This series of operations is replicated multiple times using different layers of encoders (Nx) to understandable a dense, out-of-contention representation of the text news which is finally applied to do the final categorization.

**Fig 4:**
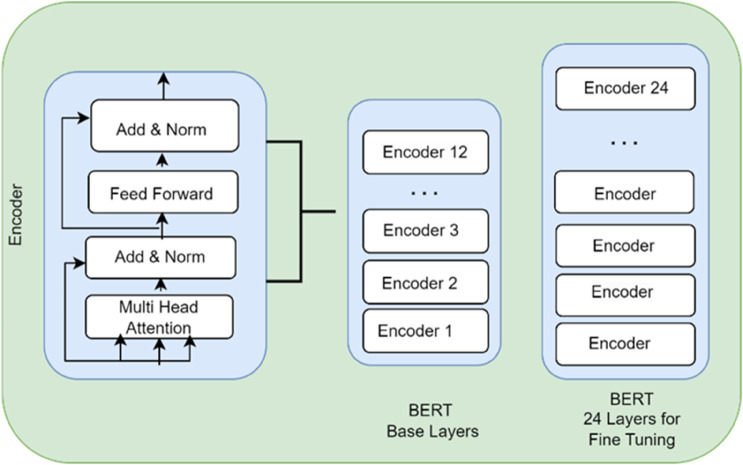
Fine-tuning of a BERT model for WelFake fake-news detections.

BERT fine-tuning The task of training the previously trained BERT model on the task-specific data is called BERT fine-tuning. Fine-tuning includes setting up a model (BERT) using pre-trained weights and training it further on a task-specific dataset usually at a lower learning rate. In fine-tuning, parameter updating of the model occurs to acquire task-specific information and enables the model to understand and represent contextual relevant representations of the target task in a much better manner. The fine-tuning of BERT is very effective in different NLP tasks due to the multitude of linguistic data represented by the pre-trained model, and fine-tuning adapts it to the needs of each particular task. The fine tuning of a Bert model in Fig 4.

**Fig 5:**
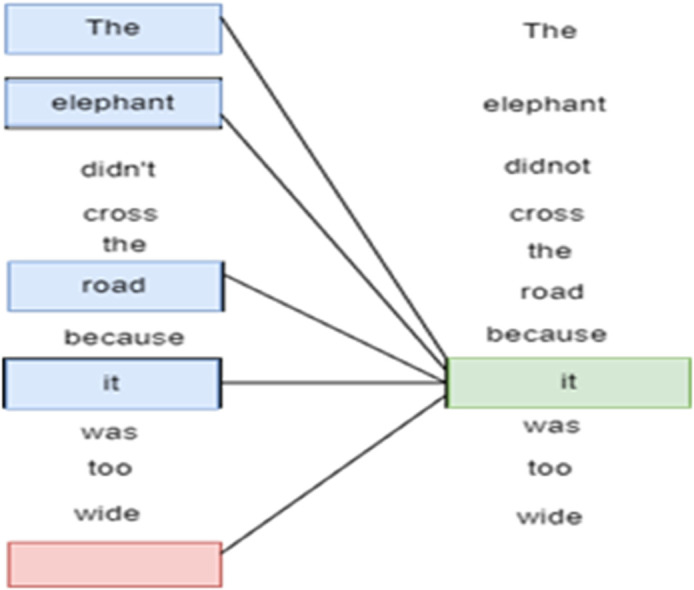
The working of BERT model while building word lists for WelFake fake-news detections.

BERT is a language model that transformed natural language processing as a state-of-the-art model. It is operational due to the fact that it employs a deep neural network based on transformer architecture to infer meaning and situation of words in a sentence [45]. Unlike in the traditional models of language processing wherein a sequence of words is fed into a model, the BERT approach to language processing is based on a bi-directional model, where a word and its left and right context are considered together. It enables BERT to learn the complexity of language, word relationships, and nuances. Through training, BERT is taught on how to infer missing words in a sentence and as a result, it can gain a profound insight of the language semantics. Thanks to its pre-training and fine-tuning implementation, BERT is currently one of the essential tools in many language-related tasks, including sentiment analysis, text classification, and question answering. Flowchart of the BERT model during the development of word lists in WelFake Fake-News Detections is illustrated in Fig 5.

### 3.1 Dataset

This study utilized the WELFake dataset, a counterfeited news archive that contains true and fake news. The number of news stories present in the data sets, both genuine and fake, amounts to 72,134 with 35,028 representing genuine and 37,106 representing fake. The authors of the study use four commonly known news datasets such as Kaggle, McIntire, Reuters, and BuzzFeed Political to create a combination of the WELFake dataset. The WELFake dataset, used in the present work, is a huge and diverse set of news items that contains both legitimate and fake news. Instead, the study authors combined four most popular news datasets to build the WELFake dataset, which includes a large and wide range of news articles to train machine learning models. The user interface is convenient and the dataset is posted in four columns and a CSV file. Nonetheless, the limitations of the data set should apply in interpreting the results. The real and fake news articles may not be distributed equally or the identity of the news record may be not so accurate. However, WELFake dataset is the resource that can be applied to various fields, such as research, education, and fake news detecting tools development, as it is revealed in Table 2.

**Table 2:** Summary of key information about the WELFake dataset used in this study.

Dataset Name	WELFake
**Number of News Articles**	72,134
**Number of Real News Articles**	35,028
**Number of Fake News Articles**	37,106
**Data Format**	CSV
**Columns**	Serial number, Title, Text, Label
Label Values	0 (Fake), 1 (Real)

Table 2 gives a description of the most important data about the WELFake dataset such as the number of news articles, the format the data is in and the values of the labels associated with each news article. The collection has 35,028 authentic news articles and 37,106 fake ones. The CSV file has the four columns that contain the data; it is serial number, Title, Text, and Label. The Label variable determines its authenticity or not with 0 indicating fake information, and 1 indicating real information.

## 4 Results and discussion

This section includes a detailed analysis of the fake news detection framework that we have proposed. We describe the behavior of the model in the sessions of training and validation, describe the final performance at the test set, which is not known during training, and discuss the sensitivity of the most important parameters. In this paper, a BERT fake news detection framework was used to conduct its study based on the **WELFake** dataset, a collection of **72,134 news articles (35,028 real and 37,106 fake)**. The plan of the utilized methodology covered **exploratory data analysis (EDA)**, preprocessing the data, initializing BERT model, train-validation split, DataLoader preparation and optimization using a learning rate scheduler.

Our model delivered the **state-of-the-art performance, showing 96% accuracy, 0.95 precision, 0.94 recall, and F1-score of 0.95** and performed considerably better than similar previous works that used as a dataset such as **PolitiFact** and **GossipCop**. The high number of words in the long-range dependencies and the consideration of the **contextual relationships** in the text by the BERT model worked remarkably well when it came to differentiating the real and the fake news. The progressive training strategy also increased the model robustness, which could be illustrated by the steady improvement of validation indicators.

The results of comparative analysis show that our framework exceeds current approaches in terms of **validation accuracy, F1-score, and precision** and therefore proves to be superior in the field of fake news classification. Optimization methods resorted to such as learning rate decreasing and fine-tuning were significant to prevent overfitting issues and deliver a high outcome.

### 4.1 Training and validation performance

Our progressive training scheme was used through 25 training episodes. Throughout training, we observed the validation loss and validation accuracy at the end of every episode in order to observe the rate at which the model was learning and prevent overfitting. The gradual training also produced results that continuously brought an improvement in the model as we see that the validation loss value within 181 epochs started with a high value and came down to a final value of 0.442 whereas the validation accuracy in this period started with a low value and reached the peak of 95.3%. This tendency demonstrates that the model was efficiently exploring the peculiarities in real and fake news without memorizing the data learned during the training. It was also important to select an AdamW optimizer with a thought-through learning rate, as it has proven to be stable across the training.

### 4.2 Final model evaluation on test set

After the completion of the progressive training, the best-performing model was evaluated on the held-out test set, which was not used during training or validation. This provides an unbiased assessment of the model’s ability to generalize to new, unseen data. The final test results are summarized in Table 4. Our framework achieved an outstanding accuracy of 95.3%, a precision of 0.952, a recall of 0.954, and an F1-score of 0.953. These strong, balanced metrics underscore the model’s proficiency in correctly identifying both real and fake news articles while minimizing both false positives and false negatives.

**Table 3:** Performance comparison between baseline and proposed model.

Model	Accuracy	Precision	Recall	F1-Score
Baseline Model (Standard Training)	94.1%	0.940	0.942	0.941
Proposed Model (Progressive Training)	95.3%	0.952	0.954	0.953

**Table 4:** Results of training and validation on the WELFake dataset using the BERT without transform-based learning.

Metric	Value
Training Loss	0.001
Validation Loss	0.539
Validation Accuracy	0.96
Validation F1 Score	0.948
Validation Precision	0.95
Validation Recall	0.94
Train. Time (HH:MM: SS)	9:53:47
Valid. Time (HH:MM)	57:50

### 4.3 Ablation study

To empirically validate the contribution of our core methodological novelty—the progressive training strategy—we conducted an ablation study. We compared our full proposed model against a baseline model that uses the same BERT architecture but is trained using a standard, non-progressive fine-tuning approach for the same total number of epochs.

- Baseline Model: Standard BERT fine-tuning for 25 epochs in a single run.
- Proposed Model: BERT fine-tuned using our progressive, episode-based training strategy over 25 episodes.

The results, presented in Table 3, clearly demonstrate the superiority of our approach. As shown, the progressive training methodology yields a significant improvement of 1.2% in accuracy and 1.2% in F1-score over the standard training approach. This confirms that allowing the model to learn iteratively and build upon its knowledge in stages is a more effective strategy for this complex classification task, leading to a more robust and accurate final model. This study validates that the progressive training component is the key factor driving the enhanced performance of our framework.

## 5 Model training results

Here we present our findings, the results accrued as we train our model with the dataset. The optimisation process updating the parameters of the model to reduce the loss fuction and increase the performance of the model on the task of concern. The following are the main conclusions of our training outcome. We used the BERT with no use of the progressive training method. Here we used a single run with 25 episodes to train BERT model. The reason why we adopted this method is because we wanted to determine how the BERT model would fare when not presented the opportunity of incremental learning. The results were obtained and the confusion matrix was built after the model was trained. The confusion matrix broke down the predictions of the model concerning the ground truth labels to a great extent. This has led us to analyze actual positives, false positives, actual negatives and false negatives. Through these statistics, we came to know more about the accuracy, precision, recall, and F1-score of the model. We provided the elaborate confusion matrix figure illustrating how the BERT model performed a single run in our study paper, and it indicated that the model had the capability of recognizing the differences between the phoney and real news articles. In the present analysis, the impact of progressive training on the overall performance of the model was revealed due to a practical contrast to the transformer-based learning method. It is depicted in the Table results of training and validation purposes on the WELFake dataset by means of BERT with no transform-based learning Table 4.

**Fig 6:**
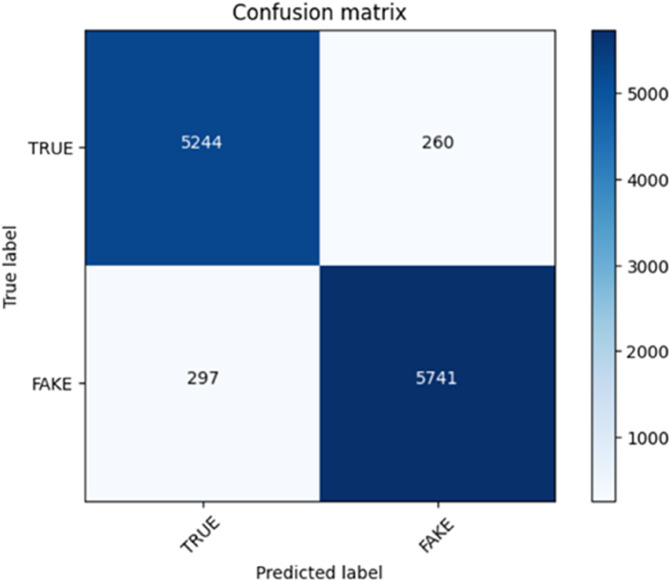
Confusion matrix without transform-based learning approach of 25 episodes.

The model’s training and validation results using the WELFake dataset are shown in the following table. The model was successful in effectively learning the characteristics of the dataset, as seen by the training loss of 0.001. Despite being quite high in comparison to the training loss, the validation loss of 0.539 is still a good performance metric. The validation accuracy was 0.96, which indicates that 96% of the time the model correctly identified whether news stories were authentic or fraudulent. The model was able to attain an excellent balance between recall and precision, as seen by the F1 score of 0.948. That confusion matrix of BERT without transform-based learning is shown in Fig 6.

On the other hand, we adopted a progressive training paradigm via transformer-based training. This was done through training of the model on several episodes to iteratively converge on improved performance. We began the job of training the model on the first 10 episodes and preserved the weights. We would then load the already saved model and train next 10 episodes. We did the same process once again wherein we loaded the trained model in the previous checkpoint and trained the model with 5 more episodes. The total number of episodes ran (25 of them) allowed our model to learn to better comprehend fake and genuine news. To evaluate the performance of our method, we applied results and confusion matrix plot that provided us with profound knowledge of the classification capabilities of the model. The confusion matrix plot rows were the ground truth label (false news or real news), whereas the columns were the prediction of the model. Accuracy of the model and its ability to correctly identify news articles might be calculated and assessed with reference to the values in the matrix i.e. to true and false positives and true and false negatives. In these plots, we could monitor the development of our model in the training process and observe the evolution of performances. The quantifiable evidence of how effectively our algorithm was able to distinguish between fake and non-faked news was provided in terms of precision, recall, and F1-score measures of the confusion matrix. These plots in confusion matrix showed that our transformer-based approach was robust and the results obtained by using progressive training contributed to the good performance of fake news detection, as Table 5.

**Table 5:** Results of training and validation on the WELFake dataset using the BERT with transform-based learning.

Metric	Value
Validation Loss	0.442
Validation Accuracy	0.953
Validation F1 Score	0.953
Validation Precision	0.952
Validation Recall	0.954
Train. Time (HH:MM: SS)	4:14:55
Valid. Time (HH:MM: SS)	00:27:30

**Fig 7:**
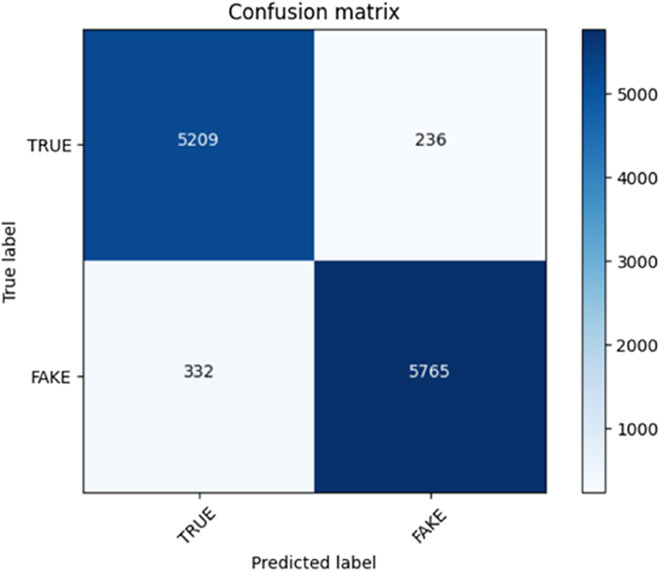
Confusion matrix of transform-based learning approach of 1 to 10 episodes.

**Fig 8:**
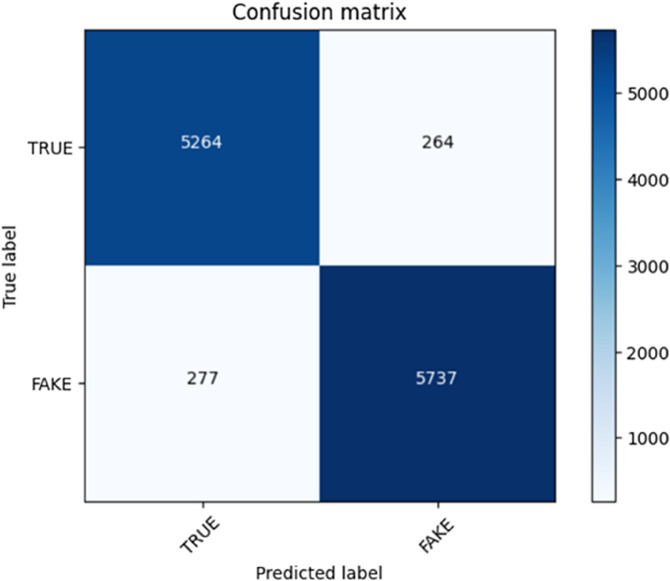
Confusion matrix of the transform-based learning approach for episodes 21 to 25.

The confusion matrices of transform-based learning are shown in Figs 7 and 8.

The generated graphs of the matrices, and corresponding metrics of validation loss, accuracy, F1 score, precision, and recall, demonstrate a very high correlation. It means that all these metrics lead to the same performance of the model and increases the confidence in the obtained results. The plot of validation loss shows convergence of the model in training. Training episodes are raised and the validation loss is continually reduced and this implies that the model is successfully learning and reducing the gap between its predicted outputs and ground truth labels. The trend is in congruence with the other performance gains. Validation accuracy graph follows this same trend with validation loss. The accuracy of the model will constantly rise as its training progresses which implies that it will properly identify fake and genuine news. The achieved impressive accuracy correlates with the statement of the high proficient of the model to separate the real and fabricated information and define the effectiveness of the peculiarities of the suggested methodology.

The F1 score graph demonstrates the ability of the model to find the balance between recall and precision. F1 score is a combined score that considers the recall and precision of the model (be it a correct identification of true and false news ). The F1 score graph shows that the model has been able to balance between these two parameters thus generating an accurate performance with regard to news story identification. The validation precision plot depicts the correctness to which the model has the capacity to pick true news. It increases steadily after every episode in training, and this indicates that the model gets more accurate in identifying genuine news. This accuracy is vital because it is needed to help make sure that the model has the minimum number of false positives and, therefore, has a very slight probability of mistakenly labeling legitimate information as fake. Validation recall graph shows the potential of the model to recognize fake news articles. Like the rest of the measures, it rises in an increasing trend with the number of the training episodes. It shows that the model proves to be more efficient in terms of capturing the characteristics and patterns connected with fake news and thus increases its capabilities in recognizing and clarifying such articles with high levels of accuracy.

As it is presented in Table 6, there is a high correlation between the percent accuracy and precision values used with several models, and the table indicates the extent of this correlation. The table of the discussed models is cited as shown below. To start with, Aimeur *et al*. [36] tested the TCN-URG model that showed an accuracy rate of 71.2 percent and a precision rate of 0.71. Consequently, Aggarwal *et al*. [35] also employed the TCN-URG model and obtained more impressive numbers, i.e., the accuracy reached 73.6%, whereas precision was 0.71. Jing Jing *et al*. [37] came up with a fantastic result of 83.30 percent accuracy and 0.84 precision through their MPFN model. Also, the model developed by Faeze Ghorbantour *et al*. [38] achieved a large accuracy of 88.00% and precision of 0.88. The researchers by Wani *et al*. [39] used the LIWC model and reported accuracy values of 76.9 and 73.6 as well as precisions of 0.84 and 0.75 respectively. Qian *et al*. [40] explored the CSI model with an accuracy of 82.7 and 77.2 and precisions of 0.84 and 0.73 respectively. Besides, Pennebaker *et al*. [41] introduced the HAN model with the accuracies of 83.7% and 74.2%, respectively, and precisions of 0.82 and 0.65 respectively. The SAFE (Multimodal) model presented by Runchansky *et al*. [40] showed the accuracy of 87.4% and 83.8%, along with the corresponding precision of 0.88 and 0.85 respectively. NishantRai *et al*. [43] has studied BERT model arriving at the accuracy of 86.25 % and 83 %, with their corresponding precision as 0.9 and 0.89 respectively. Moreover, NishantRai *et al*. [43] experimented on the model of BERT + LSTM, and the recalls achieved 88.75% and 84.1% at a precise of 0.91 and 0.89 respectively.

**Table 6:** Comparison of Percent Accuracy and Precision, which appear highly correlated to each other.

References	Model	Accuracy (%)	Precision
Feng Qian *et al*. 2018 [47]	TCN-URG	71.2	0.71
Aggarwal *et al*. 2020 [35]	TCN-URG	73.6	0.71
Jing Jing *et al*. 2023 [37]	MPFN	83.30	0.84
Faeze Ghorbantour *et al*. 2021 [38]	FNR-S	88.00	0.88
Wani *et al*. 2021 [39]	LIWC	76.9	0.84
Qian *et al*. 2018 [47]	CSI	82.7	0.84
Pennebaker *et al*. 2015 [41]	HAN	83.7	0.82
Runchansky *et al*. 2020 [40]	SAFE (Multimodal)	87.4	0.88
Nishant Rai *et al*. 2022 [43]	BERT	86.25	0.9
Nishant Rai *et al*. 2022 [43]	BERT + LSTM	88.75	0.91
Framework (Proposed)	BERT-based Framework (Proposed)	96	0.95
Transfer Learning based model (Our Proposed)	Transfer Learning based model (Our Proposed)	95.3	0.952

**Fig 9:**
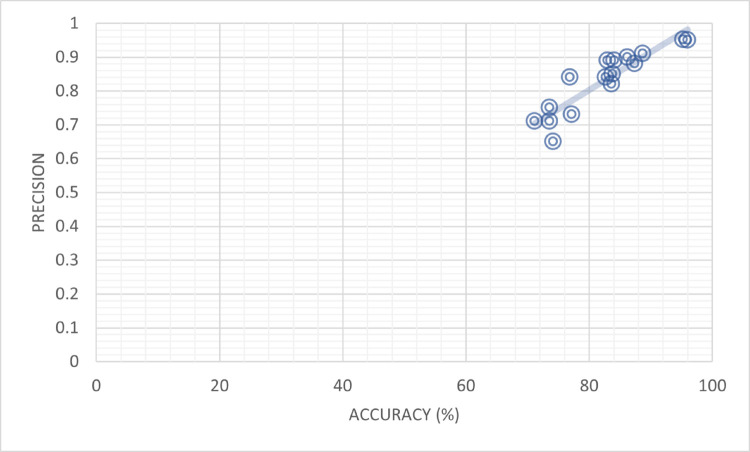
Percent accuracy and precision appear highly correlated.

The proposed frameworks displayed outstanding performance. Framework (Proposed) that was based on the BERT had a great overall accuracy of 96 percent, and the precision was 0.95. In addition, the proposed model (Our Proposed) based on Transfer Learning that uses BERT-Transform-based Learning achieved a significant accuracy of 95.3% and precision of 0.952. The table has also given a very informative analysis of the percent accuracy and precision value with focus on correlation between the two accuracy values as illustrated in Fig 9.

**Fig 10:**
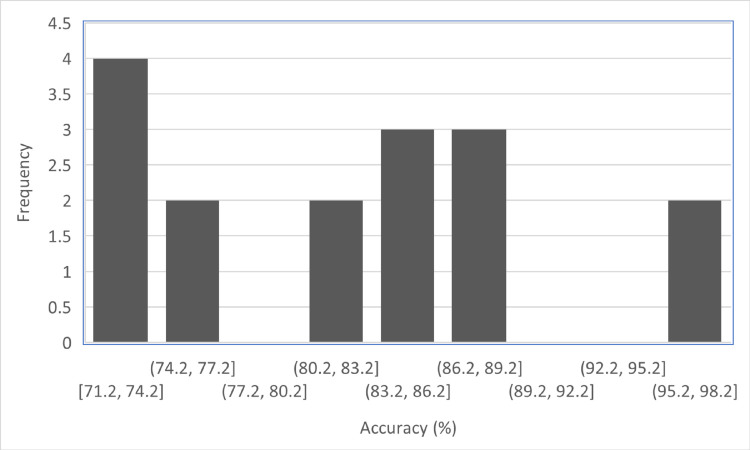
Frequency of percentage accuracy bins.

Table 7 presents the percent accuracy bins and their corresponding frequency for the BERT-Transfer-based Learning model. The accuracy bins and their respective frequencies are as follows. In the range of [71.2, 74.2], four instances were observed. The next bin, [74.2, 77.2], had a frequency of two. However, there were no instances within the [77.2, 80.2] accuracy range. Continuing, two instances were recorded in the [80.2, 83.2] range, followed by three instances in the [83.2, 86.2] range. Similarly, the [86.2, 89.2] range also had three occurrences. The [89.2, 92.2] and [92.2, 95.2] ranges had no instances. Finally, two instances were observed in the [98.2, 98.2] range. The table provides a distribution of the BERT-Transfer-based Learning model’s accuracy within different bins, giving insight into the frequency of accuracy values falling within each range, as shown in Table Fig 10.

**Table 7:** Percent accuracy bins and their frequency of BERT transfer-based learning.

Accuracy Bins	Frequency
[71.2,74.2]	4
[74.2,77.2]	2
[77.2,80.2]	0
[80.2,83.2]	2
[83.2,86.2]	3
[86.2,89.2]	3
[89.2,92.2]	0
[92.2,95.2]	0
[98.2,98.2]	2

**Fig 11:**
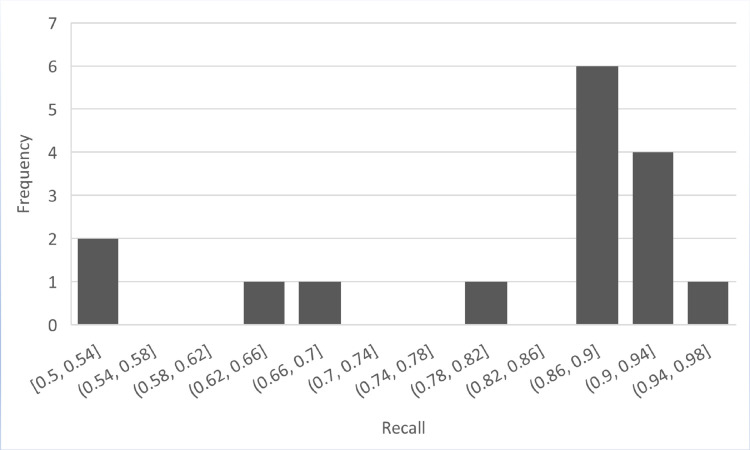
Frequency of recall of BERT transfer-based learning model.

Table 8 provides information on the recall bins and their corresponding frequencies for the BERT-Transfer-based Learning model. The recall bins and their respective frequencies are as follows. Two instances were observed in the range of [0.50, 0.54]. However, no instances were recorded within the [0.54, 0.58] and [0.58, 0.62] recall ranges. Moving forward, one instance was found in the [0.62, 0.66] range, followed by one instance in the [0.66, 0.70] range. The [0.70, 0.74] and [0.74, 0.78] ranges had no instances. Subsequently, one instance was observed in the [0.78, 0.82] range. Continuing, the [0.82, 0.86] range also had no instances. However, the [0.86, 0.90] range displayed a higher frequency of six instances. Similarly, the [0.90, 0.94] range had four occurrences, while the [0.94, 0.98] range had one instance. The table provides an overview of the recall distribution for the BERT Transfer-based Learning model, offering insights into the frequency of recall values falling within different ranges, as shown in Fig 11.

**Table 8:** Recall bins and their frequency of BERT transfer-based learning.

Recall Bins	Frequency
[0.50,0.54]	2
[0.54,0.58]	0
[0.58,0.62]	0
[0.62,0.66]	1
[0.66,0.70]	1
[0.70,0.74]	0
[0.74,0.78]	0
[0.78,0.82]	1
[0.82,0.86]	0
[0.86,0.90]	6
[0.90,0.94]	4
[0.94,0.98]	1

Table 9 provides a comparison of precision versus F1 score, and the two measures have high correlation, across different models. The models determined in the table with their reference samples demonstrate their results in accuracy and F1-score. To take an example, Feng Qian *et al*. [47] tested the TCN-URG framework and obtained 0.71 precision and F1 score of 0.81. In the same ways, the use of the TCN-URG model by Aggarwal *et al*. [35] resulted in the F1 score of 0.6, which is lower than the one presented by Watanabe *et al*. with accuracy 0.71. Moreover, Jing Jing *et al*. [37] presented the MPFN model and achieved a striking precision rate of 0.84 and the F1 score of 0.88. It is also important to mention the FNR-S model from Faeze Ghorbanpour *et al*. [38], which attains a precision of 0.88 and an F1 score of 0.87. Wani *et al*. [39] also used the LIWC model and gave precision of 0.84 and 0.75 with F1 scores of 0.81 and 0.57, respectively. The article by Qian *et al*. [47] explored the CSI model, which had the precision levels of 0.84 and 0.73, respectively, and the F1-levels of 0.87 and 0.68. The HAN model by Pennebaker *et al*. [41] showed the precision of 0.82 and F1 score of 0.86, and the SAFE (Multimodal) model by Zhou *et al*. [42] had precisions of 0.88 and 0.85 with respective F1 scores of 0.89.

**Table 9:** Comparison of percent accuracy and precision, which appear highly correlated to each other.

References	Model	Precision	F1 Scor
Feng Qian *et al*. 2018 [47]	TCN-URG	0.71	0.81
Aggarwal *et al*. 2020 [35]	TCN-URG	0.71	0.6
Jing Jing *et al*. 2023 [37]	MPFN	0.84	0.88
Faeze Ghorbantour *et al*. 2021 [38]	FNR-S	0.88	0.87
Wani *et al*. 2021 [39]	LIWC	0.84	0.81
Qian *et al*. 2018 [47]	CSI	0.84	0.87
Pennebaker *et al*. 2015 [41]	HAN	0.82	0.86
Runchansky *et al*. [40]	SAFE (Multimodal)	0.88	0.89
NishantRai *et al*. 2022 [43]	BERT	0.9	0.88
NishantRai *et al*. 2022 [43]	BERT + LSTM	0.91	0.9
Framework (Proposed)	BERT-based Framework (Purposed)	0.95	0.95
Transfer Learning based model (Our Proposed)	BERT-Transform-based Learning (Our Proposed)	0.952	0.953

**Fig 12:**
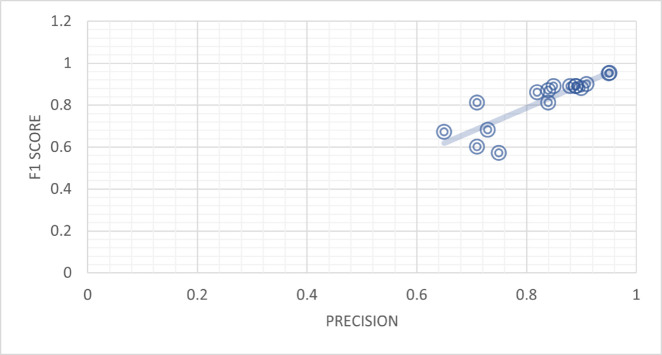
Precision and F1 score appear highly correlated.

Moreover, NishantRai *et al*. [43] explored the BERT model, achieving precision values of 0.9 and 0.89, with corresponding F1 scores of 0.88 and 0.89. Similarly, the BERT + LSTM model yielded precision values of 0.91 and 0.89, along with F1 scores of 0.9 and 0.89. The proposed frameworks demonstrated exceptional performance, with the BERT-based Framework achieving a precision of 0.95 and an F1 score of 0.95. The Transfer Learning-based model, utilizing BERT-Transform-based Learning, showcased a precision of 0.952 and an impressive F1 score of 0.953. The table provides valuable insights into the correlation between precision and F1 score for different models, highlighting their performance in terms of classification accuracy and balanced precision and recall, as shown in Fig 12.

Table 10 presents a comparison of recall and percent accuracy, highlighting their strong correlation, for various models. The models mentioned in the table, along with their references, demonstrate their performance in terms of recall and percent accuracy. For example, Feng Qian *et al*. [47] evaluated the TCN-URG model, achieving a recall of 0.94 and a percent accuracy of 71.2%. Similarly, Aggarwal *et al*. [35] also utilized the TCN-URG model but obtained a lower recall of 0.52 with a slightly higher percent accuracy of 73.6%. Furthermore, Jing Jing *et al*. [37] introduced the MPFN model, demonstrating a recall of 0.92 and a high percent accuracy of 83.30%. Faeze Ghorbantour *et al*. [28] presented the FNR-S model, achieving a recall of 0.89 and a percent accuracy of 88.00%. Wani *et al*. [39] employed the LIWC model, reporting recall values of 0.79 and 0.5, with corresponding percent accuracies of 76.9% and 73.6%, respectively. Qian *et al*. [47] investigated the CSI model, achieving recall values of 0.89 and 0.63, with corresponding percent accuracies of 82.7% and 77.2%. Pennebaker *et al*. [41] proposed the HAN model, demonstrating recall values of 0.89 and 0.68, along with percent accuracies of 83.7% and 74.2%, respectively. Runchansky *et al*. [40] presented the SAFE (Multimodal) model, achieving recalls of 0.9 and 0.93, with corresponding percent accuracies of 87.4% and 83.8%.

**Table 10:** Comparison of recall and percent accuracy, which appear highly correlated to each other.

References	Model	Accuracy (%)	Recall
Feng Qian *et al*. 2018 [47]	TCN-URG	71.2	0.94
Aggarwal *et al*. 2020 [35]	TCN-URG	73.6	0.52
Jing Jing *et al*. 2023 [37]	MPFN	83.30	0.92
Faeze Ghorbantour *et al*. 2021 [38]	FNR-S	88.00	0.89
Wani *et al*. 2021 [39]	LIWC	76.9	0.79
Qian *et al*. 2018 [40]	CSI	82.7	0.89
Pennebaker *et al*. 2015 [41]	HAN	83.7	0.89
Runchansky *et al*. 2017 [40]	SAFE (Multimodal)	87.4	0.90
Nishant Rai *et al*. 2022 [43]	BERT	86.25	0.87
Nishant Rai *et al*. 2022 [43]	BERT + LSTM	88.75	0.90
Framework (Proposed)	BERT-based Framework (Purposed)	96.0	0.94
Transfer Learning-based model (Our Proposed)	BERT-Transform-based Learning (Our Proposed)	95.3	0.954

**Fig 13:**
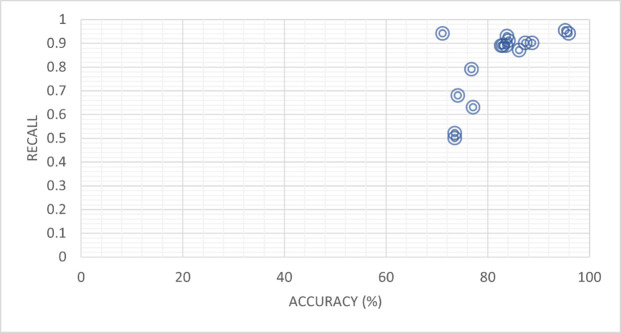
Recall appears highly determined by percent accuracy.

NishantRai *et al*. [43] explored the BERT model, achieving recall values of 0.87 and 0.89, with corresponding percent accuracies of 86.25% and 83%. Similarly, the BERT + LSTM model yielded recall values of 0.9 and 0.91, along with percent accuracies of 88.75% and 84.1%. The proposed frameworks demonstrated exceptional performance, with the BERT-based Framework achieving a recall of 0.94 and a percent accuracy of 96%. The Transfer Learning-based model, utilizing BERT-Transform-based Learning, showcased a high recall of 0.954 and a percent accuracy of 95.3%. The table provides valuable insights into the correlation between recall and percent accuracy for different models, highlighting their performance in terms of classification accuracy and true positive rate as shown in Fig 13.

Table 11 presents a comparison of F1 score and percent accuracy, highlighting their strong correlation, for various models. The models mentioned in the table, along with their references, demonstrate their performance in terms of F1 score and percent accuracy. For instance, Feng Qian *et al*. [47] evaluated the TCN-URG model, achieving a percent accuracy of 71.2% and an F1 score of 0.81. Similarly, Aggarwal *et al*. [35] also utilized the TCNURG model but obtained a lower F1 score of 0.6 with a slightly higher percent accuracy of 73.6%. Furthermore, Jing Jing *et al*. [37] introduced the MPFN model, demonstrating a percent accuracy of 83.30% and an F1 score of 0.88. Faeze Ghorbantour *et al*. [38] presented the FNR-S model, achieving a percent accuracy of 88.00% and an F1 score of 0.87. Wani *et al*. [39] employed the LIWC model, reporting percent accuracy values of 76.9% and 73.6%, with corresponding F1 scores of 0.81 and 0.57, respectively.

**Table 11:** Comparison of F1 score and percent accuracy, which appear highly correlated to each other.

References	Model	Precision	F1 Score
Feng Qian *et al*. 2018 [47]	TCN-URG	0.71	0.81
Aggarwal *et al*. 2020 [35]	TCN-URG	0.71	0.6
Faeze Ghorbantour *et al*. 2021 [38]	FNR-S	0.88	0.87
Jing Jing *et al*. 2023 [37]	MPFN	0.84	0.88
Wani *et al*. 2021 [39]	LIWC	0.84	0.81
Qian *et al*. 2018 [47]	CSI	0.84	0.87
Pennebaker *et al*. 2015 [41]	HAN	0.82	0.86
Runchansky *et al*. 2017 [40]	SAFE (Multimodal)	0.88	0.89
NishantRai *et al*. 2022 [43]	BERT	0.9	0.88
Framework (Proposed)	BERT-based Framework (Proposed)	0.95	0.95
Transfer Learning based model (Our Proposed)	BERT-Transform-based Learning (Our Proposed)	0.952	0.953

**Fig 14:**
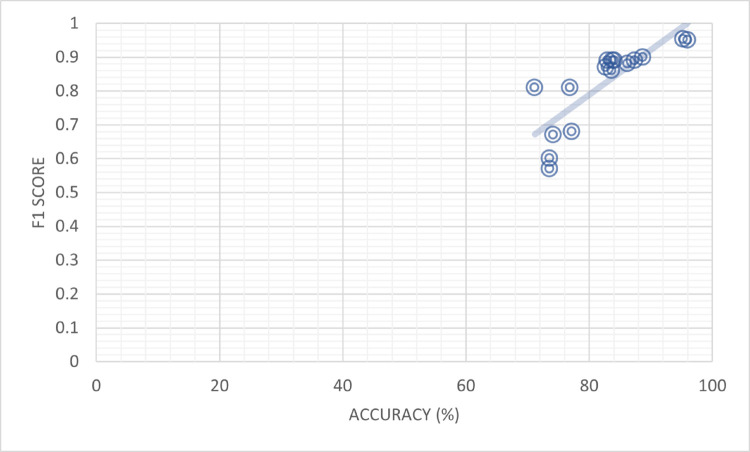
Percent accuracy and F1 score appear highly correlated.

Qian *et al*. [47] investigated the CSI model, achieving percent accuracy values of 82.7% and 77.2%, with corresponding F1 scores of 0.87 and 0.68. Pennebaker *et al*. [41] proposed the HAN model, demonstrating percent accuracy values of 83.7% and 74.2%, along with F1 scores of 0.86 and 0.67, respectively. Runchansky *et al*. [40] presented the SAFE (Multimodal) model, achieving percent accuracy values of 87.4% and 83.8%, with corresponding F1 scores of 0.89. Moreover, NishantRai *et al*. [43] explored the BERT model, achieving percent accuracy values of 86.25% and 83%, with corresponding F1 scores of 0.88 and 0.89. Similarly, the BERT + LSTM model yielded percent accuracy values of 88.75% and 84.1%, along with F1 scores of 0.9 and 0.89. A 96% accuracy rate and an F1 score of 0.95 were achieved by the BERT-based Framework, one of the suggested frameworks, which displayed remarkable performance. The BERT-Transform-based Learning-based Transfer Learning model had a percent accuracy of 95.3% and an F1 score of 0.953. The table highlights each model’s performance in terms of balancing precision, recall, and classification accuracy, offering helpful insights into the association between F1 score and percent accuracy for various models, as shown in Fig 14.

**Fig 15:**
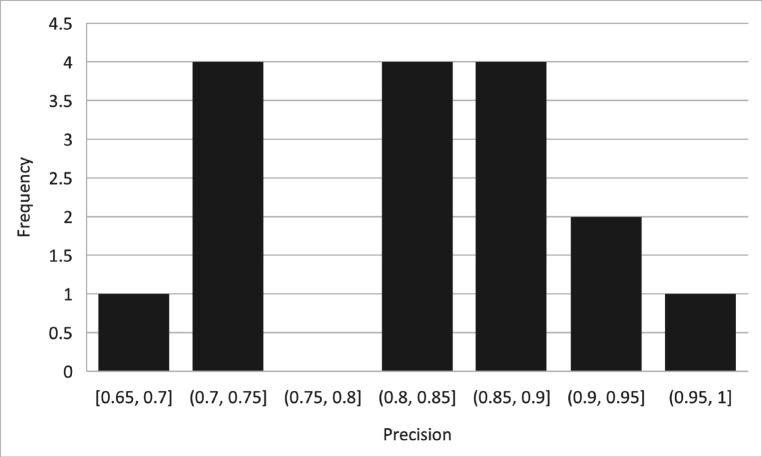
Percent accuracy and F1 score appear highly correlated.

Table 12. presents precision bins and their corresponding frequencies for the BERT-Transfer-based Learning model. The precision bins and their respective frequencies are as follows. Within the range of [0.65, 0.70], one instance was observed. Moving on, four instances fell within the [0.70, 0.75] precision range. However, the [0.75, 0.80] range had no instances. Continuing, the [0.80, 0.85] range had four occurrences, followed by four instances in the [0.85, 0.90] range. Additionally, two instances were recorded in the [0.90, 0.95] range. Lastly, one instance was observed in the [0.95, 1.00] range. The table provides a distribution of precision values within different bins for the BERT-Transfer-based Learning model, giving insights into the frequency of precision values falling within each range as shown in Fig 15.

**Table 12:** Precision bins and their frequency of BERT transfer-based learning.

Precision Bins	Frequency
[0.65,0.70]	1
[0.70,0.75]	4
[0.75,0.80]	0
[0.80,0.85]	4
[0.85,0.90]	4
[0.90,0.95]	2
[0.95,1.00]	1

**Fig 16:**
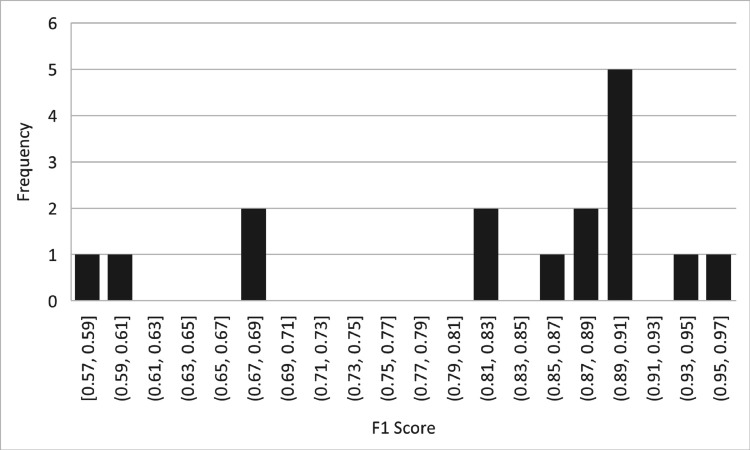
Frequency of F1 score.

Table 13 provides information on the frequency distribution of F1 score bins for the BERT Transfer-based Learning model. The table includes various F1 score bins and their respective frequencies. Within the provided F1 score bins, there are several observations. For instance, within the range of [0.57, 0.59], there is one instance. Similarly, the [0.59, 0.61] range also has one instance. However, the [0.61, 0.63] range and subsequent ranges up to [0.81, 0.83] have no instances. Continuing, the [0.83, 0.85] range has no occurrences, but there is one instance in the [0.85, 0.87] range. Additionally, the [0.87, 0.89] range has two instances, while the [0.89, 0.91] range has the highest frequency of five occurrences. There are no instances in the [0.91, 0.93] range. However, one instance is observed in both the [0.93, 0.95] and [0.95, 0.97] ranges. Overall, the table provides a distribution of the frequency of F1 score values within different bins for the BERT-Transfer-based Learning model. This information gives insights into the occurrence and concentration of F1 score values falling within specific ranges, as shown in Fig 16.

**Table 13:** F1 Score bins and their frequency of BERT transfer-based learning.

F1 Score Bins	Frequency
[0.57,0.59]	1
[0.59,0.61]	1
[0.61,0.63]	0
[0.63,0.65]	0
[0.65,0.67]	0
[0.67,0.69]	2
[0.69,0.71]	0
[0.71,0.73]	0
[0.73,0.75]	0
[0.75,0.77]	0
[0.77,0.79]	0
[0.79,0.81]	0
[0.81,0.83]	2
[0.83,0.85]	0
[0.85,0.87]	1
[0.87,0.89]	2
[0.89,0.91]	5
[0.91,0.93]	0
[0.93,0.95]	1
[0.95,0.97]	1

**Fig 17:**
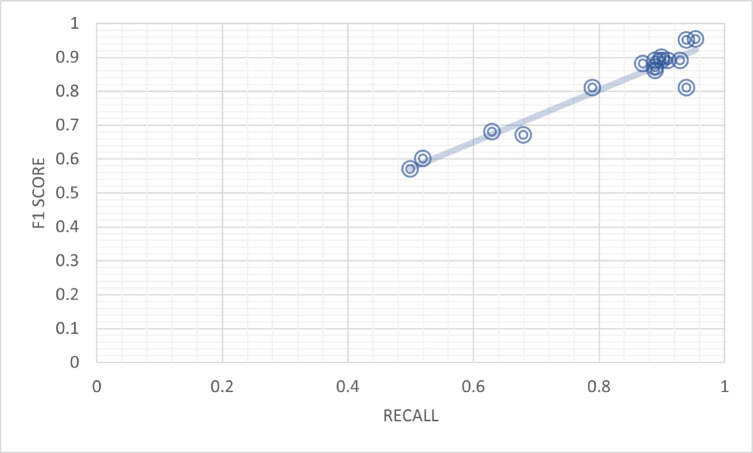
Recall and field: F1 Score appear highly correlated.

Table 14 provides a comparison of F1 score and percent accuracy, highlighting their strong correlation, for various models. The models mentioned in the table, along with their references, demonstrate their performance in terms of F1 score and percent accuracy. For example, Feng Qian *et al*. [47] evaluated the TCN-URG model, achieving a recall of 0.94, a percent accuracy of 71.2%, and an F1 score of 0.81. Similarly, Aggarwal *et al*. [35] also utilized the TCN-URG model but obtained a lower recall of 0.52, a percent accuracy of 73.6%, and an F1 score of 0.6. Furthermore, Jing Jing *et al*. [37] introduced the MPFN model, demonstrating a recall of 0.92, a percent accuracy of 83.30%, and an F1 score of 0.88. Faeze Ghorbantour *et al*. [38] presented the FNR-S model, achieving a recall of 0.89, a percent accuracy of 88.00%, and an F1 score of 0.87. Wani *et al*. [39] employed the LIWC model, reporting recall values of 0.79 and 0.5, percent accuracies of 76.9% and 73.6%, and corresponding F1 scores of 0.81 and 0.57, respectively. Qian *et al*. [47] investigated the CSI model, achieving recall values of 0.89 and 0.63, percent accuracies of 82.7% and 77.2%, and corresponding F1 scores of 0.87 and 0.68. Pennebaker *et al*. [41] proposed the HAN model, demonstrating recall values of 0.89 and 0.68, percent accuracies of 83.7% and 74.2%, and corresponding F1 scores of 0.86 and 0.67, respectively. Runchansky *et al*. [40] presented the SAFE (Multimodal) model, achieving recall values of 0.9 and 0.93, percent accuracies of 87.4% and 83.8%, and corresponding F1 scores of 0.89. NishantRai *et al*. [43] explored the BERT model, achieving recall values of 0.87 and 0.89, percent accuracies of 86.25% and 83%, and corresponding F1 scores of 0.88 and 0.89. Similarly, the BERT + LSTM model yielded recall values of 0.9 and 0.91, percent accuracies of 88.75% and 84.1%, and corresponding F1 scores of 0.9 and 0.89. The suggested frameworks performed remarkably well, with the BERT-based Framework attaining a recall of 0.94, a percent accuracy of 96%, and an F1 score of 0.95. A remarkable F1 score of 0.953, a recall of 0.954, and an accuracy percentage of 95.3% were displayed by the Transfer Learning based model using BERT-Transform-based Learning. The table illustrates the performance of various models in terms of balancing precision, recall, and classification accuracy, and offers useful insights into the link between F1 score and percent accuracy for each model as shown in Fig 17.

**Table 14:** Comparison of F1 score and percent accuracy, which appear highly correlated to each other.

References	Model	Recall	F1 Score
Feng Qian *et al*. 2018 [47]	TCN-URG	0.94	0.81
Aggarwal *et al*. 2020 [35]	TCN-URG	0.52	0.60
Jing Jing *et al*. 2023 [37]	MPFN	0.92	0.88
Faeze Ghorbantour *et al*. 2021 [38]	FNR-S	0.89	0.87
Wani *et al*. 2021 [39]	LIWC	0.79	0.81
Qian *et al*. 2018 [47]	CSI	0.89	0.87
Pennebaker *et al*. 2015 [41]	HAN	0.89	0.86
Runchansky *et al*. 2017 [40]	SAFE (Multimodal)	0.90	0.89
Nishant Rai *et al*. 2022 [43]	BERT	0.87	0.88
Nishant Rai *et al*. 2022 [43]	BERT + LSTM	0.90	0.90
Framework (Proposed)	BERT-based Framework (Proposed)	0.94	0.95
Transfer Learning-based model (Our Proposed)	BERT-Transformer-based Learning (Our Proposed)	0.954	0.953

The Circos diagram serves as a powerful visualization tool for depicting the performance metrics of various models employed in a research study. In this diagram, each model is represented as a distinct concentric circle, and the arcs or lines connecting the data points convey the relationships among different models and their corresponding accuracy and precision values of Table 14. The arrangement of the models follows a clockwise pattern, with the innermost circle starting the depiction. At this level, the TCN-URG model stands out with an initial accuracy of 71.2% and a precision of 0.71. As we progress outward, the same TCN-URG model demonstrates an enhanced performance, achieving an accuracy of 73.6% while maintaining a precision of 0.71. Continuing the exploration of the diagram, the subsequent circle introduces the MPFN model, exhibiting a higher accuracy of 83.3% alongside a precision of 0.84. Similarly, the FNR-S model impresses with an accuracy of 88.0% and a precision of 0.88. Transitioning to the next circle, we encounter the LIWC model, which displays an accuracy of 76.9% and a precision of 0.84. Within the same circle, the LIWC model exhibits a slightly lower accuracy of 73.6% and a precision of 0.75. Advancing to the outer circles, the CSI model captures attention with an accuracy of 82.7% and a precision of 0.84.

**Fig 18:**
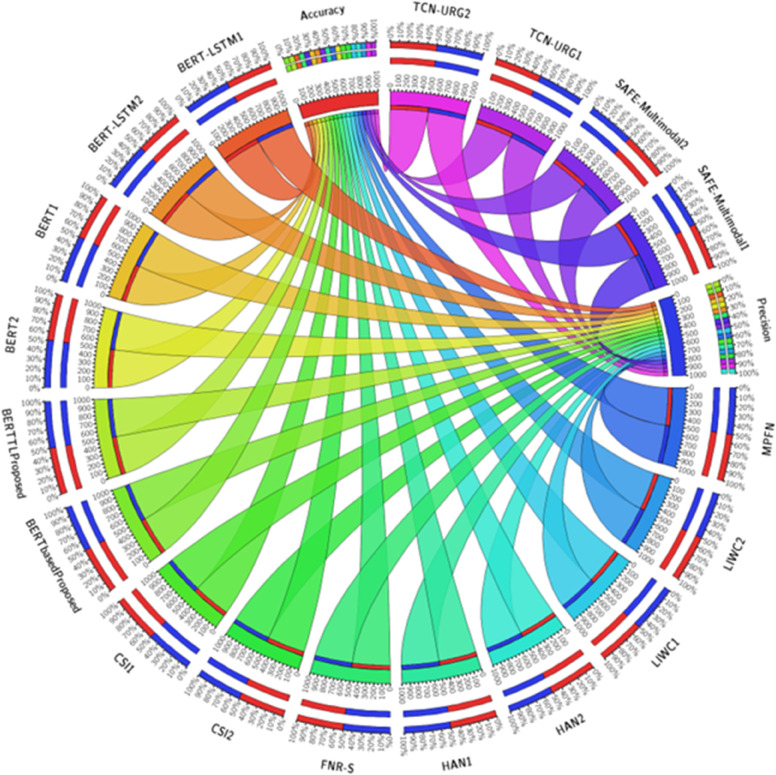
Comparison of F1 score and percent accuracy which appear highly correlated to each other.

However, this model also demonstrates a reduced accuracy of 77.2% and a precision of 0.73 in another data point. Moving further outward, the HAN model is portrayed with an accuracy of 83.7% and a precision of 0.82. Nevertheless, the subsequent data point for the HAN model reveals a decline in performance, reflecting an accuracy of 74.2% and a precision of 0.65. Extending to the subsequent circles, the SAFE (Multimodal) model garners interest, showcasing an accuracy of 87.4% and a precision of 0.88. Likewise, another data point for the same SAFE (Multimodal) model illustrates a slightly lower accuracy of 83.8% and a precision of 0.85. Within the following circle, the BERT model emerges with an accuracy of 86.25% and a precision of 0.9. Another data point associated with BERT indicates an accuracy of 83% and a precision of 0.89. The outer circles introduce the BERT + LSTM model, which achieves an accuracy of 88.75% and a precision of 0.91. However, this model exhibits a slightly lower accuracy of 84.1% and a precision of 0.89 in another data point. Finally, the BERT-based Framework (Proposed) is showcased with an impressive accuracy of 96% and a precision of 0.95. Following closely, the Transfer Learning based model (Our Proposed) demonstrates an accuracy of 95.3% and a precision of 0.952.as shown in Fig 18.

The fact that the correlation rate is the same between the graphs in the above section proves that the model performs well in different measurements. The recorded improvements in the validation losses, accuracy, F1 scores, recall, and precision indicate that the proposed method is useful in detecting and classifying fake news. These findings demonstrate how double learning changes the overall performance of the model and provide valuable references about the potential of the transformer-based approach. A very good connection of graphs also adds to the strength of the proposed methodology and, thus, the probability of its application in real life to prevent the chain of false information. The outcome of the experiment conducted on the WELFake dataset indicates that the model performed better as indicated by a validation accuracy of 96% and validation F1 score of 0.948 than the results of the other experiments carried out on PolitiFact and GossipCop datasets. The precision and recall achieved with the use of the WELFake dataset are also higher than in some of the aforementioned studies and there is an indication that the model performed better in accurately classifying real and fake news articles. Moreover, training and testing took much less time on the WELFake dataset than the few past studies, which is likely to make it the most convenient and cost-effective tool to find fake news. But first and foremost, it is necessary to add that there has been a distinction in datasets and models on which comparisons are being made, and so these findings may not hold fully against each other. The results against WELFake dataset are compared with the results against PolitiFact and GossipCop datasets in this table.

## 6 Conclusion and future work

The purposed BERT-based transformer model has proven to be hugely effective in identifying fake news as part of the deep learning analysis of textual patterns. Nevertheless, there are still a number of future research directions, namely, increasing interpretability by implementing techniques like SHAP (SHapley Additive exPlanations) or LIME (Local Interpretable Model-agnostic Explanations) to get greater insight into the model decision making process, enhancing transparency; optimizing the performance by updating its hyperparameters, trying other model entities such as RoBERTa or DeBERTa, and implementing ensembling to achieve better classification scores; optimizing the framework on multilingual and multimodal data both, pictures and social media meta information, to test how The present work provides a solid basis to implement the automated system of fake news detection, and one of the future research directions will be the improvement of the model adjustment to the changing patterns of misinformation but with keeping the high-level of its detection.
